# Stereotactic ablative radiotherapy for acquired resistance to EGFR therapy in metastatic non-small cell lung cancer

**DOI:** 10.3389/fonc.2022.1092875

**Published:** 2023-01-16

**Authors:** Rodolfo Chicas-Sett, Juan Castilla Martinez, Abrahan Hernández Blanquisett, Juan Zafra, Jorge Pastor-Peidro

**Affiliations:** ^1^ Department of Radiation Oncology, ASCIRES GRUPO BIOMEDICO, Valencia, Spain; ^2^ Department of Medical Oncology, Centro Hospitalario Serena del Mar, Cartagena, Colombia; ^3^ Group of Translational Research in Cancer Immunotherapy, Health and Medical Research Center (CIMES), Institute of Biomedical Research in Malaga (IBIMA), Malaga, Spain; ^4^ Department of Radiation Oncology, Virgen de la Victoria University Hospital, Malaga, Andalusia, Spain; ^5^ Faculty of Medicine, University of Malaga, Malaga, Andalusia, Spain

**Keywords:** SAbR, EGFR - epidermal growth factor receptor, NSCLC - lung adenocarcinoma - EGFR - ALK - BRAF - KRAS - RET - MET - PD-L1 - ROS1, oligoprogressive disease, targeted therapy, metastatic NSCLC, oligometastatic

## Abstract

The advent of targeted therapy has transformed the treatment paradigm and survival of patients with metastatic non-small cell lung cancer (NSCLC) with driver mutations. The development of acquired resistances during treatment with tyrosine kinase inhibitors (TKIs) impedes a prolonged survival in many patients. This fact is leading to the use of locally ablative therapies such as stereotactic ablative radiotherapy (SABR) to counter these resistances. SABR is a non-invasive treatment that can be delivered in multiple locations and has already proven effective in oligometastatic disease. Clinical evidence suggests that the combination of SABR with TKIs prolongs progression-free survival (PFS) in metastatic NSCLC patients with mutations in epidermal growth factor receptor (EGFR), with international guidelines recommending their use in unfavorable scenarios such as oligoprogressive disease. In this publication, we have reviewed the available evidence on EGFR-TKIs resistance mechanisms and the combination of SABR with TKI in metastatic NSCLC with EGFR mutations. We also describe the utility and clinical recommendations of this combination in oligometastatic and oligoprogressive disease.

## Introduction

Historically, stage IV non-small cell lung cancer (NSCLC) has been considered an incurable disease, only candidate for palliative treatments. Chemotherapy (CT), the standard treatment, offered survival rates of less than a year (1). More recently, the identification of several genetic variants with a predomination of mutations in the oncogene for epidermal growth factor receptor (EGFR) has transformed the therapeutic landscape in this subset of patients (2–5). Targeted therapy (TT) with tyrosine kinase inhibitors (TKIs) has reported median overall survivals (OS) of 22-36 months in patients with an EGFR mutation (6). However, the onset of primary and acquired resistances continue to be the main obstacle to further improve these results (7). The main example is the T790M mutation, which is responsible for 60% of TKI resistance in this setting (8). Moreover, third-generation TKIs are also affected by the C797S mutation, causing resistance in 10-26% of patients receiving osimertinib as second-line and 7% when used as first-line (9).

Stereotactic ablative radiotherapy (SABR) is an effective treatment for oligometastatic disease (10). Recently, several studies have reported results on its promising combination with immunotherapy and TT (11–13). Various clinical guidelines recommend the use of SABR in oligometastatic or oligoprogressive patients with driver mutations (14). This has garnered a special interest in the potentially decisive role of SABR in the eradication tumor clones that are resistant to systemic therapy by extending clinical benefit. In this review, we aim to present the clinical data that justify the use of SABR as a therapeutic agent that may counteract the resistance to EGFR-TKI in patients with metastatic NSCLC.

## State of targeted therapy in EGFR-mutated NSCLC

EGFR mutations occur in 12% of NSCLC cases, although Asian patients have a higher prevalence at 47% (15). The majority of these patients have no prior history of smoking, and the most common activating mutations are deletions in exon 19 and the L858R point mutation in exon 20.

Currently, EGFR testing is recommended for all patients with stage IB-IV adenocarcinoma-type NSCLC and stage IV squamous cell carcinoma. EGFR inhibitors are the preferred treatment for NSCLC patients with somatic EGFR mutations. Those stage IB-IIIA EGFR+ patients initially treated with surgery and achieving a complete resection (R0) may benefit from adjuvant treatment with osimertinib 80 mg daily for 3 years following the results of the phase 3 ADAURA trial, which showed a substantial benefit in terms of disease-free survival. This benefit was most evident in stage IIIA patients. In this group, at 24 months, 88% of patients treated with adjuvant osimertinib were alive and disease-free compared to 32% in the placebo group. This trial reported that osimertinib had an acceptable safety profile with serious adverse events occurring in 16% of patients versus 13% in the placebo group (16).

Stage IV disease remains incurable, but EGFR inhibitors have shown consistent clinical and statistically significant benefits for the EGFR+ population compared with standard CT. Erlotinib, gefitinib, afatinib, dacomitinib and osimertinib are effective as monotherapy, while combination regimens such as erlotinib plus ramucirumab or erlotinib plus bevacizumab have emerged as alternative treatments for selected patients (17–23). Subsequently, the FLAURA trial has reported better results for osimertinib compared to gefitinib or erlotinib in terms of PFS and OS (21). It should be noted that these EGFR inhibitors are also effective in patients with less common EGFR mutations, such as S7681, L861Q and G719X, because these mutations are also sensitive to these treatments (24). However, recent real-world data suggest that osimertinib has better outcomes in patients with common EGFR mutations compared to patients harboring these rare mutations (25).

Ultimately, there is strong evidence from phase 3 randomized trials to support the use of EGFR inhibitors as first-line treatment for stage IV disease, and as adjuvant treatment after complete resection for stage IB-IIIA, being osimertinib the currently preferred option. Questions about the best regimen after failure to osimertinib in both settings warrant future research.

## Mechanisms of resistance to EGFR-TKIs: “failure patterns”

Although EGFR inhibitors have represented a turning point in the treatment of EGFR-mutated lung cancer, most stage IV patients will suffer from disease progression and eventually die from it. Lung cancer is a heterogeneous disease and sensitivity to treatment is not the same for all EGFR mutations. The best example is EGFR exon 20 insertion mutations, as most variants do not respond to EGFR inhibitors. Amivantamab (a bispecific EGFR-MET) and mobocertinib (an irreversible kinase inhibitor), are under investigation for these patients. Both have shown promising results in recent trial and expected to the widely available in the future (26, 27).

On the other hand, many patients who initially respond to EGFR inhibitors will develop secondary resistances through different mechanisms. The first-generation drugs erlotinib and gefitinib are reversible inhibitors, while second and third generation agatinib, dacomitinib and osimertinib are irreversible inhibitors. For first and second-generation inhibitors most cases will develop a new mutation in EGFR, the most frequent being T790M. This mutation produces a biochemical change in the ATP levels required to achieve a half-maximal reaction rate, ultimately resulting in a sensitivity to inhibitors which is similarly low as in EGFR wild–type (28). Osimertinib provides clinical and statistical benefit for patients who develop this resistance and is the preferred option for this subgroup (29). Another resistance mechanism called “bypass” is based on alternative molecular pathways that activate proliferation and survival independent of EGFR activation, the most frequent being the amplification of the ERBB2 and MET genes. Mutations in other genes such as BRAF, PIK3CA, KRAS, PTEN, NF-1 have also been described but are less frequent (28). Ongoing trials are testing several drugs (capmatinib, sotarasib, trastuzumab-deruxtecan and trastuzumab-emtasine) that are already used in clinical practice as a second line option for lung cancer and their benefit is not limited to the population with EGFR mutation (29–33).

A great challenge for oncologists nowadays is the acquired resistance to the third-generation inhibitor Osimertinib. The mutation in C797S is the most frequent in this context and most patients retain the T790M mutation after progression (6). There is currently no consensus on the standard treatment after failure to osimertinib. Rebiopsy is recommended at the time of relapse, either in tissue or liquid biopsy. Combinations of immunotherapy with CT, antiangiogenic agents or combinations of TT can be employed, with no choice being clearly superior. Clinical trials will hopefully establish the standard therapy for these progressing patients in the coming years.

## SABR in metastatic NSCLC

The standard treatment for patients with metastatic NSCLC is systemic therapy based on CT and/or immunotherapy. In the case of NSCLC with driver mutations, TT is the treatment of choice. However, although improved from only CT regimes, median survival is still limited. When analyzing the relapse patterns of these patients, most recurrences occur at the same sites of the initial metastatic disease. Considering this fact, the addition of SABR in oligometastatic patients or those with low tumor burden with ECOG ≤2 could improve PFS and OS (34).

Even though no phase III trials that validate these results have been published, the use of SABR in clinical practice is increasing, with even international guidelines such as the National Comprehensive Cancer Network (NCCN) recommending the addition of ablative therapy in *de novo* oligometastatic patients to try to improve survival and in oligoprogressive disease to prolong the benefit of systemic therapy (35).

These recommendations are based on the results of different retrospective and prospective studies. As for retrospective data, a meta-analysis published in 2014 including 757 oligometastatic patients (1-5 lesions but 96.5% ≤2 metastases) treated with local therapy (surgery +/- radiotherapy/SABR). Median PFS was 11 months, with a two-year PFS of 25%. Furthermore, Median OS was 26 months and two-year OS was 51.1%. These are relevant numbers, especially considering that CT was part of the first-line treatment in only 17.7% of cases (36). A more recent meta-analysis of 693 NSCLC patients (78% oligometastatic) compared local consolidative treatment (surgery or radiotherapy) vs systemic therapy. In the oligometastatic subgroup, hazard ratio (HR) for PFS was 0.30 (p<0.001) and 0.41 (p<0.001) for OS, which shows the benefit of local therapy in these patients (37).

Moreover, several phase II studies have presented results that further show the effectivity of SABR in oligometastatic NSCLC:

- A phase II study conducted in Belgium analyzed 26 patients with ≤5 metastases diagnosed by positron emission tomography (PET) receiving CT, or SABR (5 Gy in 10 fractions) in patients who were not candidates for CT. Seventy five percent of patients presented synchronous oligometastases. With a median follow-up of 16.4 months, median PFS was 11.2 months and 1-year PFS was 45%. In terms of OS, median was 23 months and 67% at one year. Local failure in the irradiated sites was only 15% (38).
- Iyengar et al. published in 2017 a single-institution cohort of patients with ≤6 metastases (including the primary tumor) treated with at 4-6 cycles of CT and who were not treatable with TT for driver mutations. Patients who did not progress after CT were randomized to SABR plus maintenance CT vs CT alone. After 29 patients and a median follow-up of 9.6 months, the study was closed due to the interim analysis showing positive results, with a median PFS of 9.6 months in the SABR group vs 3.5 months in the control group (p=0.01) (13).
- The OLIGOMEZ study (2019) is a multicenter randomized study of patients with up to three metastases (>90% synchronous) after induction systemic therapy (CT or TT in case of EGFR or ALK+). Forty-nine patients which did not progress after systemic therapy were randomized to receive local therapy (surgery and/or radiotherapy/SABR) or maintenance systemic therapy (17% received no treatment in this arm). After a median follow-up of 38.8 months, median PFS was 14.2 months in the local treatment group vs 4.4 months in the control group (p=0.022). Median OS was also significantly better in the experimental arm (41.2 months vs 18.9, p=0.017). An interesting endpoint analyzed in the study was the time until the diagnoses of new lesions, which showed a non-statistically significant tendency in favor of the local treatment group (14.2 vs 6 months). This could suggest that local treatment could even limit the dissemination potential of the disease (12, 13, 39).
- The final results of the SABR-COMET trial were published in 2020. This study included 99 patients with different primary tumors (18 patients had lung cancer) and with 1-5 metastases (93 ≤3 lesions) that were randomized 2:1 to receive SABR plus best palliative treatment vs palliative treatment alone). With a median follow-up of 51 months, median PFS was 11.6 months with SABR vs 5.4 in the control arm (p=0.001), and four-year PFS was 21.6% vs 3.2%. A favorable impact of SABR was also reported in terms of OS, with a median of 50 months vs 28 months. After excluding prostate cancer from the analysis, OS remained better for SABR (five-year OS 33.1% vs 16.2%, p=0.085) (10). In 2022, updated results have been published with a median follow-up of 5.7 years. Eight-year PFS was 21.3% in the SABR arm vs 0% in the control arm (p<0.001). The positive impact of SABR was also observed in eight-year OS: 27.2% vs 13.6% (p=0.008). These recent data supports the durability of the effect of SABR (34).

In terms of toxicity, all these studies confirm that SABR is a safe approach. Recently, a phase II multicenter study has analyzed toxicity in 381 patients treated with SABR, reporting grade ≥3 toxicity of 4% and grade ≥2 of 8% (40).

All the studies above justify the use of SABR in oligometastatic patients (≤5) with ECOG ≤2 that do not progress to systemic therapy. However, it must be noted that these are studies with few patients, with a heterogeneous systemic therapy and none of them include the use of immunotherapy, which currently has a key role in these patients. We are awaiting the results of phase III studies in oligometastatic NSCLC such as NRG-LU-002 (NCT03137771), SARON (NCT02417662), and SABR-COMET 3 (NCT03862911) and 10 (NCT03721341). Many of these are aiming to demonstrate the benefit in OS of the addition of SABR to systemic therapy. Moreover, the OMEGA (NCT03827577) study is comparing locally ablative treatment vs conventional systemic therapy (41).

Given these results, and while ongoing studies report their data, SABR may be recommended in oligometastatic patients (with up to 3-5 metastases) with ECOG ≤2 that do not progress to initial systemic therapy. The ESTRO-ASTRO consensus also adds that size is not a limiting factor and that, in fact, larger lesions can be treated with adequate constraints (42).

## SABR + TKI

### Rationale of the combination

The biological effect of SABR differs from conventional radiotherapy. SABR unleashes additional microvascular damage through the activation of different cellular pathways, producing a higher rate of tumor cell death (43). Several studies have reported that the combination of SABR and TKIs could have a synergistic effect. This could be justified by the following ideas: a) EGFR-mutated patients present a higher sensitivity to radiation (44), b) TKIs inhibit DNA repair (45), c) there is a reduction in T790M mutations after irradiation (46).

EGFR+ patients tend to suffer from disease progression 1-2 years after the star of TKIs. In fact, 60% develop acquired resistance explained by the T790M mutation T790M (18, 20, 47), and 70% in the form of oligoprogression (48). Yajing Wu et al. found it a recent meta-analysis that adding local therapy to systemic treatment improves PFS and OS with no increase in grade ≥3 toxicity (37). These reasons have led to the considering SABR as a tool that may revert these resistances with the following objectives:

-Lower/maintain disease burden.
-Delay disease progression.
-Extend the duration of clinical Benefit from TKIs, mainly in the oligometastatic scenario (oligorrecurrence, oligopersistence and oligoprogression).
-Local control of symptomatic lesions.

### Evidence

Prospective studies evaluating the combination of SABR and TKIs are limited and focus on more unfavorable scenarios such as oligoprogressive disease. While most evidence comes from retrospective data, results are similar to those reported in controlled studies, showing a positive impact on PFS (**Table 1**). At present, clinical guidelines like the ones by NCCN recommend SABR in oligoprogressive EGFR+ NSCLC patients (35).

**Table 1:** Selected studies of SABR and TKIs combination in metastatic NSCLC EGFR-mutated patients.

Author	Study design	N	Strategy	Fractionation	TKI	Timing	Local control (%)	PFS (median, mo)	OS (median, mo)	Grade ≥ 3 toxicity (%)
Wang et al. [2022] SINDAS Trial (49)	Phase III Randomized	133	SABR + TKI TKI	25-40 Gy/5 fx	Gefitinib, Erlotinib, Icotinib	Concurrent	91.2 55.4 (p<0.001)	20.2 12.5 (p<0.001)	25.5 17.6 (p<0.001)	35.4 41.4
Peng et al. [2019] (50)	Phase II Randomized	61	TKI + SABR TKI	NR	NR	Sequential	NR	17.4 10.3 (p=0.042)	NR	0
Iyengar et al. [2014] (13)	Phase II	24	SABR + TKI	27-33 Gy/3 fx 35-40 Gy/5 fx 10-20 Gy/1fx	Erlotinib	Concurrent	93.6 at 9 mo	14.7	20.4	8.3
Wang et al. [2021] (51)	Retrospective	308	SABR + TKI TKI	70 Gy/10 fx 60 Gy/8 fx 50 Gy/5 fx	Gefitinib, Erlotinib, Icotinib, Osimertinib, Afatinib	Concurrent	NR	19.4 13.7 (p=0.034)	34.5 43.5 (p=0.557)	20 17.8 (p=ns)
Santarpia et al. [2020] (52)	Retrospective	36	SABR +TKI	12-60 Gy/2-30 fx SABR (36%); RT (64%)	Gefitinib	Concurrent	NR	6.3	38.7	2.7
Kroeze et al. [2021] (53)	Retrospective	65 (EGFR=49)	SABR+TKI	20 Gy/1fx Median BED 95.3 Gy/3fx	Gefitinib, Osimertinib, Afatinib, Erlotinib	Concurrent	84 at 24 mo	10.4	181	6.2
Pembroke et al. [2018] (54)	Retrospective	163 Various tumors	SABR + TKI	BED 42-150 Gy	NR	Concurrent (44.3%) Sequential (55.7%)	73	11	31	0.6
Chan et al. [2017] (55)	Retrospective	50	SABR + TKI CT	50-60 Gy/3 fx 35 Gy/5 fx 24-35 Gy/2-5 fx	Osimertinib	Sequential	100	7 4.1 (p=0.0017)	28.2 14.7 (p=0.026)	4
Conforti et al. [2013] (56)	Retrospective	15	SABR + TKI	NR	Erlotinib, Gefitinib	Concurrent	NR	10.9	39	0
Yu et al. [2013] (57)	Retrospective	18	TKI + SABR/surgery	NR	Gefitinib,Erlotinib	Sequential	NR	10	41	11.1
Weickhardt et al. [2012] (58)	Retrospective	25 (EGFR=10)	SABR or surgery	40 Gy	Crizotinib, Erlotinib	Sequential	NR	6.2	NR	0

SABR, Stereotactic Ablative Radiotherapy; TKI, Tyrosine Kinase Inhibitor; CT, Chemotherapy; Gy, Gray; Fx, fraction; PFS, Progression-free survival; OS, Overall Survival; NR, Not Reported; Mo: Months; BED, Biologically Effective Dose; EGFR, epidermal grown factor receptor.

The study by Weickhardt et al. was one of the first to evaluate the use of SABR to mitigate the resistance to TKIs. Of 65 patients included, 27 presented EGFR mutation and were in progression to TKIs. Despite the limited size sample, there was a benefit in PFS for patients who received SABR and maintained the line of TKIs (58). Further retrospective studies have also reported a tendency towards better PFS for patients treated with SABR, ranging from 6.2 to 19.4 months (**Table 1**). In the context of oligoprogression, maintaining TKIs after SABR has shown better PFS (7 vs 4.1 months) and OS (28.2 vs 14.7 months) compared to CT (55).

Although most studies included oligometastatic patients (up to 5 lesions), Kroeze et al. did actually assess polymetastatic patients. This study in particular found a promising PFS of 10.4 months despite 75% of patients presenting brain metastases (53).

The upfront combination of SABR with TKIs at the start of therapy was evaluated by Iyengar et al. in a phase II trial (13). This study found a PFS of 14.7 months and OS of 20.4 months. A posterior phase II randomized study found a PFS of 17.4 vs 10.3 months in the systemic therapy alone group (50). Finally, the phase III SINDAS trial included 133 oligometastatic EGFR+ patients treated with upfront SABR plus TKI vs TKI alone. A statistically significant benefit was observed in the combination group, with an OS of 25.5 months vs 17.4 months (p<0.001) (49).

### Is it safe to combine SABR and TKIs? What should the sequence be?

Generally, the combination of SABR and TKIs has reported safe outcomes. Randomized studies such as SINDAS found no significant different in grade ≥3 toxicity between the combination and TKI alone (49). **Table 1** shows that grade ≥3 side effects are acceptable in most studies. However, liver SABR and treatment with sorafenib has reported a high risk of gastrointestinal toxicity (59). The combination of osimertinib and thoracic radiotherapy has reported a potential increase in pulmonary toxicity. Nonetheless, these data come from retrospective studies with small cohorts, and percentages are similar to those of CRT-induced pneumonitis (60).

In terms of the treatment sequence, there is no clear consensus on whether the combination should be administered concurrently or if TKI should be interrupted during SABR. Studies that have administered this combination concurrently have not reported high grade ≥3 toxicities (0-11%) (13, 47). However, more data are needed to establish the most adequate approach.

### SABR dose and fractionation

The optimal dose and fractionation of SABR when combined with TKIs is still unknown. Although the studies in **Table 1** are very heterogeneous, doses have generally followed recommendations for definitive treatments with SABR alone, trying to reach biologically equivalent doses (BED) ≥ 100 Gy.

## Future directions

Ongoing clinical trials are aiming to confirm the data reported up to this point. In oligoprogression to TKIs, the phase II/III HALT trial (NCT03256981) will include an experimental arm in which patients will receive SABR to up to three extracranial sites while continuing TKI. In an earlier scenario, the randomized NORTHSTAR trial (NCT03410043) will evaluate if the combination of SABR plus osimertinib is better than osimetinib alone in stage IIIB/IV EGFR+ NSCLC as first-line therapy.

A pending challenge to overcome is the incorporation of translational aspects into clinical practice through novel biomarkers. Detection of circulating DNA can predict treatment response, but also detect possible resistances (61). The use of these blood biomarkers in clinical trials combining SABR and TKIs could be promising as their implementation into clinical practice would be easily accessible.

## Conclusion

SABR is a safe and effective approach for the treatment of oligometastatic NSCLC. Moreover, it can be used in multiple locations and does not require the interruption of systemic therapy. Acquired resistance to TKIs is a challenging scenario in which the use of SABR has reported very promising results at overcoming the resistance mechanisms to TKIs in metastatic NSCLC. Although evidence remains limited, clinical benefit has outweighed the risks mainly in oligoprogresive disease. In this setting, TKIs maintenance after successful SABR has prolonged PFS and, in some cases, OS. The combination has also reported an effective role as first line, although the best strategy is still unknown and warrants further research.

## Author contributions

RC-S- Conception and design, collection of data, data analysis and interpretation, manuscript writing, final approval of manuscript JCM- Conception and design, collection of data, manuscript writing, final approval of manuscript JZ- Conception and design, data analysis and interpretation, manuscript writing, final approval of manuscript AH- Conception and design, collection of data, manuscript writing, final approval of manuscript JP-P - Conception and design, collection of data, manuscript writing, final approval of manuscript. All authors contributed to the article and approved the submitted version.
